# Supplementary material for: Responsivity of Two Pea Genotypes to the Symbiosis with Rhizobia and Arbuscular Mycorrhiza Fungi—A Proteomics Aspect of the “Efficiency of Interactions with Beneficial Soil Microorganisms” Trait
Source: Int J Mol Sci. 2025 Jan 8;26(2):463. doi: 10.3390/ijms26020463 (PMC11764919; doi:10.3390/ijms26020463)
Supplement: Supplementary file 1 [file ijms-26-00463-s001.zip › Supplementary Information 1_revized.pdf]

Responsivity of two pea genotypes to the symbiosis with rhizobia and arbuscular mycorrhiza fungi – a proteomics aspect of the "Efficiency of Interactions with Beneficial Soil Microoganisms" trait

Supplementary information 1

Andrej Frolov [1,]*, Julia Shumilina [1], Sarah Etemadi Afshar [2], Valeria Mashkina [3], Ekaterina Rhomanovskaya [3], Elena Lukasheva [3], Alexander Tsarev [3], Anton S. Sulima [4], Oksana Y. Shtark [4], Christian Ihling [2], Alena Soboleva [1,]*, Igor A. Tikhonovich [4,5] and Vladimir A. Zhukov [3,4]*

[1] Laboratory of Analytical Biochemistry and Biotechnology, K.A. Timiryazev Institute of Plant Physiology Russian Academy of Science: frolov@ifr.moscow (A.F.); oriselle@yandex.ru (A.S); schumilina.u@yandex.ru (J.S.)

[2] Institute of Pharmacy, Martin-Luther Universität Halle-Wittenberg; christian.ihling@pharmazie.uni-halle.de (C.I.)

[3] St. Petersburg State University; elena_lukasheva@mail.ru (E.L.)

[4] All-Russia Research Institute for Agricultural Microbiology; asulima@arriam.ru (A.S.S.), oshtark@arriam.ru (O.S.), i.tikhonovich@arriam.ru (I.A.T)

* Correspondence: frolov@ifr.moscow (A.F.); vladimir.zhukoff@gmail.com (V.Z.)

Directory

Tables

Table S1-1 Protein extraction yields, protein concentrations in extracts and optical densities of individual SDS-PAGE lanes corresponding to individual samples of protein extracts isolated from *Pisum sativum* root cells of intact plants, plants in complex with nodule bacteria, plants in complex with nodule bacteria and arbuscular mycorrhiza, plants treated with mineral nutrition………………..….3

Table S1-2 Chromatographic and mass spectrometric parameters used for the nanoUHPLC-ESI-LIT-Orbitrap-DDA-MS experiments…………………………………………………………………..4

Table S1-3 Parameters for database search settings…………………………………………….....6

Figures

Figure S1-1. Electrophoregrams of 5 μg total protein isolates from root cells of *Pisum sativum* plants……………………………………………………………………………………………7

Figure S1-2. Electrophoregrams of 5 μg total protein isolates tryptic hydrolysates from root cells of *Pisum sativum* plants……………………………………………………………………………8

Figure S1-3 Numbers of peptides (A), proteins (B) and protein groups (C), identified *Pisum sativum* (genotype K-8274) plants……………………………………………………………………………9

Figure S1-4 Numbers of peptides (A), proteins (B) and protein groups (C), identified *Pisum sativum* (genotype K-3358) plants………………………………………………………………………...10

Figure S1-5 Functional annotation of the proteins, which are more abundant in the roots of the pea k-3358 and k-8274 plants……………………………………………………………………………..11

Figure S1-6 Functional annotation of the proteins identified as differentially abundant in the roots of pea (*P.sativum*, genotype k-3358) plants grown without soil complements and in presence of mineral salts supplemented to the soil……………………………………………………………………….12

Figure S1-7 Functional annotation of the proteins identified as differentially abundant in the roots of pea (*P.sativum*, genotype k-8274) plants grown without soil complements and in presence of mineral salts supplemented to the soil……………………………………………………………………….13

Figure S1-8 Functional annotation of the proteins identified as differentially abundant in the roots of pea (*P.sativum*, genotype k-3358) plants grown without soil complements and in presence of rhizobial bacteria supplemented to the soil (after formation of legume-rhizobial symbiosis, NB)……………..14

Figure S1-9 Functional annotation of the proteins identified as differentially abundant in the roots of pea (*P.sativum*, genotype k-3358) plants grown without soil complements and in presence of rhizobial bacteria and arbuscular micorrhiza fungi supplemented to the soil (after formation of legume-rhizobial symbiosis and arbuscular micorrhiza, combined inoculation, AMF+NB)……………………………15

Figure S1-10 Functional annotation of the proteins identified as differentially abundant in the roots of pea (*P.sativum*, genotype k-8274) plants grown without soil complements and in presence of rhizobial bacteria and arbuscular micorrhiza fungi supplemented to the soil (after formation of legume-rhizobial symbiosis and arbuscular micorrhiza, combined inoculation, AMF+NB)……………………………16

Tables

Table S1-1 Protein extraction yields, protein concentrations in extracts and optical densities of individual SDS-PAGE lanes corresponding to individual samples of protein extracts isolated from *Pisum sativum* root cells of intact plants, plants in complex with nodule bacteria, plants in complex with nodule bacteria and arbuscular mycorrhiza, plants treated with mineral nutrition

Sample	Sample weight (g)	Protein concentration (mg/mL)	Protein recovery (mg/g fresh weight)	Optical densities
Control_8274	248.7	8.7	3.5	19100
Control_8274	248.4	4.2	1.7	19300
Control_8274	253.7	5.7	2.2	18500
Control_3358	245.3	8.4	3.4	18700
Control_3358	253.0	5.2	2.1	19200
Control_3358	249.1	6.0	2.4	18200
NB_8274	252.7	4.9	1.9	16858
NB_8274	244.1	4.8	2.0	17110
NB_8274	250.4	3.8	1.5	17026
NB_3358	253.5	6.8	2.7	17865
NB_3358	246.9	4.8	1.9	18116
NB_3358	256.4	4.5	1.8	17529
AMF+NB 8274	244.1	3.2	1.3	17611
AMF+NB 8274	248.1	3.6	1.5	17611
AMF+NB 8274	252.6	4.9	1.9	16877
AMF+NB 3358	248.1	5.2	2.1	16388
AMF+NB 3358	251.3	3.5	1.4	17121
AMF+NB 3358	252.8	3.9	1.5	18507
Mineral nutrition 8274	244.2	3.8	1.6	16359
Mineral nutrition 8274	251.3	4.0	1.6	15450
Mineral nutrition 8274	248.7	2.2	0.9	15450
Mineral nutrition 3358	249.0	4.2	1.7	16194
Mineral nutrition 3358	252.8	3.1	1.2	16111
Mineral nutrition 3358	256.0	3.8	1.5	15368

Table S1-2 The conditions of nanoHPLC separation and the settings for nano-electrospray ionization-Orbitrap mass spectrometry (nanoESI-Orbitrap-MS) applied for the analysis of pea seed tryptic peptides using nLC 1000 nanoflow chromatography system (Thermo Fisher Scientific, Bremen, Germany) equipped with EASY-Spray™ Source and coupled online to LTQ-Orbitrap Velos Pro hybrid mass spectrometer (Thermo Fisher Scientific, Bremen, Germany)

Chromatography

**Parameter**	**Settings**
Trap column	PepMap 100 C18 trap column (300 µm ×5 mm, 3 µm particle size, Thermo Fisher Scientific, Bremen, Germany)
Analytical column	PepMap RSLC C18 column (75 µm ×250 mm, 2 µm particle size, Thermo Fisher Scientific, Bremen, Germany)
Injection volume	10 µl
Sample pickup flow	20 µl min^{-1}
Eluents	A: 0.1% (*v/v*) aq. formic acid;
	B: 0.1% (*v/v*) formic acid in acetonitrile
Elution flow rate	0.3 µl min^{-1}
Column temperature	40ºC
Elution regimen	Linear gradient - from 3 to 25% eluent B in 170 min
	Linear gradient – from 25 to 35% eluent B in 10 min
	Linear gradient – from 35 to 80% eluent B in 5 min
	Isocratic at 80% eluent B during 10 min
Isocratic flow	Isocratic 75% eluent B till the start of the next run
Trap column equilibration	12 µl of A at 250 bar
Analytical column equilibration	3 µl of A at 250 bar

MS conditions

Ionization mode	Positive
Resolution	60 000
Ion spray voltage (IS)	1900 V
Capillary temperature	275 °C
Default charge state	2

Microscans	1
AGC Target	$3x10^{-6}$
Maximum IT	100 ms
Number of scan ranges	1
Mass to charge ratio (*m/z*) range	300 – 1500
Spectrum data type	Profile
**MS/MS and data-dependent acquisition (DDA) conditions**	
Fragmentation	Collision activated dissociation
Microscans	1
Resolution	17 500
AGC target	$5x10^{-4}$
Maximum IT	50 ms
Loop count	10
MSX count	1
TopN	5
Isolation window	2 m/z
Isolation offset	0 m/z
Scan range	Automatic
Fixed first mass	120 *m/z*
Normalized collision energy	35 V
Spectrum data type	Centroid
Minimum AGC target	$1.5x10^{3}$
Intensity threshold	$3x10^{4}$
Charge state rejected	Unassigned, +1
Peptide match	Preferred
Exclude isotopes	On
Duration of dynamic exclusion duration	60 s

Table S1-3 Database search settings employed for peptide identification and protein annotation in pea roots

Database search settings	
Analysis program	SEQUEST
Protease	Trypsin
Missed cleavage sites	3
FDR	0.05
MS mass tolerance	0.8 ppm
MS/MS mass tolerance	20 pm

Figures

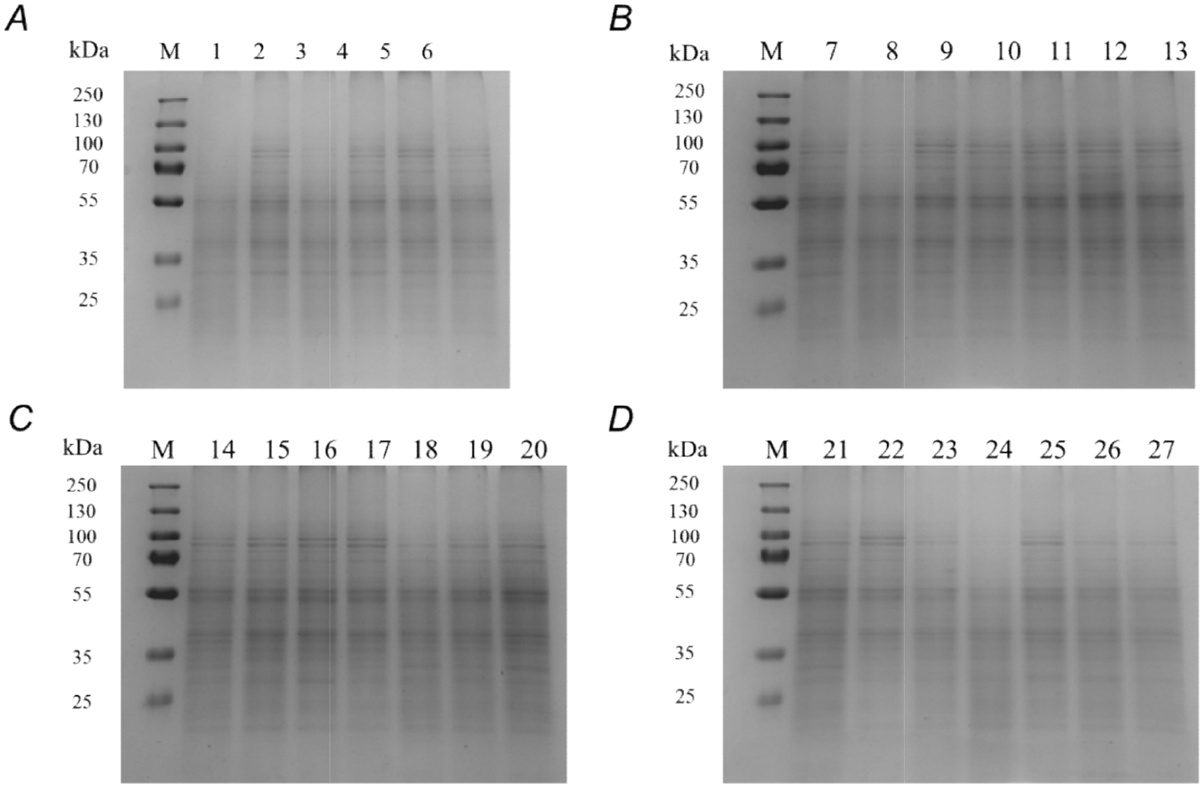

Figure S1-1 Electrophoregrams of the total protein isolates (5 µg aliquots loaded on each lane) obtained from the *P. sativum* (genotypes k-3358 and k-8274) roots (lanes 1-27) of the plants grown in the absence of mineral nutrition and supplementation of symbiotic organisms (A), of the plants inoculated with rhizobia with formation of root nodules (B), of the plants inoculated with arbuscular micorrhiza fungi and nodule bacteria with formation of micorrhiza and root nodules (C) and of the plants grown in the presence of mineral nitrogen source and absence of microorganisms (D). M – marker of molecular weight PageRuler™ Plus Prestained Protein Ladder, 10 to 250 kDa, #26619 (Thermo Scientific). The gels were stained with 0.1% (w/v) Coomassie G-250 solution.

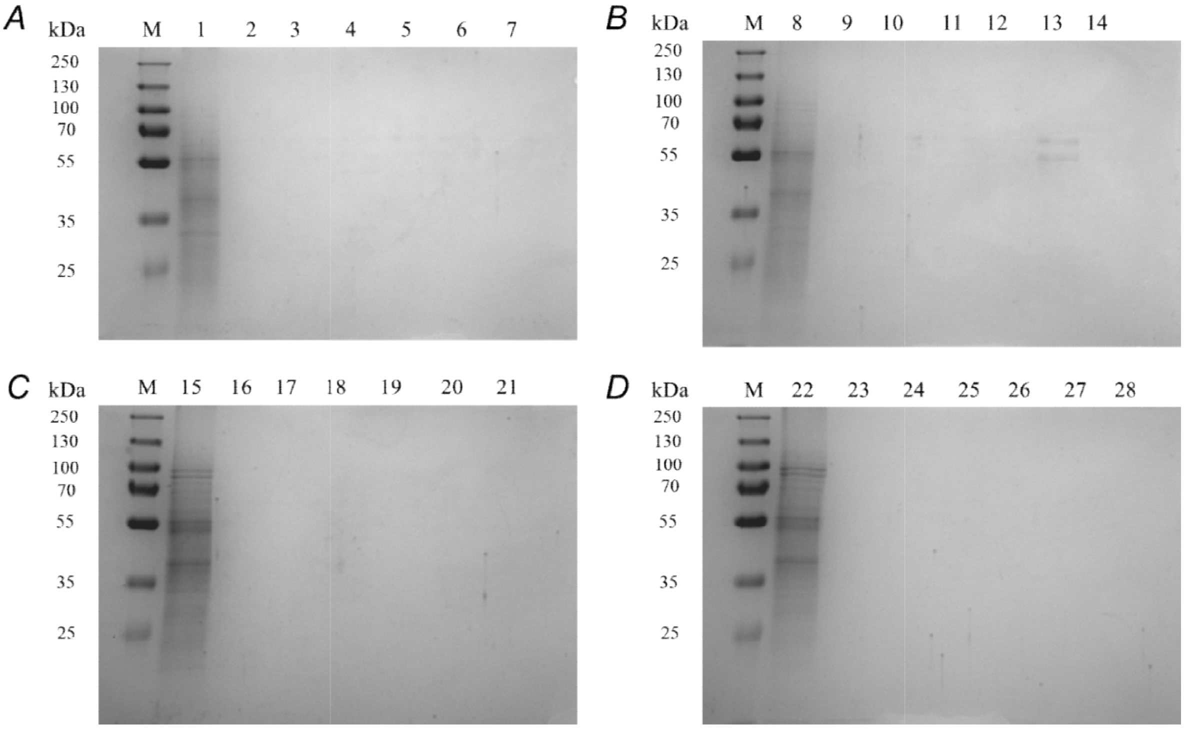

Figure S1-2 Electrophoregrams of the tryptic hydrolysates of total protein isolates (5 µg aliquots) obtained from the *P. sativum* (genotypes k-3358 and k-8274) roots (lanes 1-27) of the plants grown in the absence of mineral nutrition and supplementation of symbiotic organisms (A), of the plants inoculated with rhizobia with formation of root nodules (B), of the plants inoculated with arbuscular micorrhiza fungi and nodule bacteria with formation of micorrhiza and root nodules (C) and of the plants grown in the presence of mineral nitrogen source and absence of microorganisms (D). M – marker of molecular weight PageRuler™ Plus Prestained Protein Ladder, 10 to 250 kDa, #26619 (Thermo Scientific). The gels were stained with 0.1% (w/v) Coomassie G-250 solution.

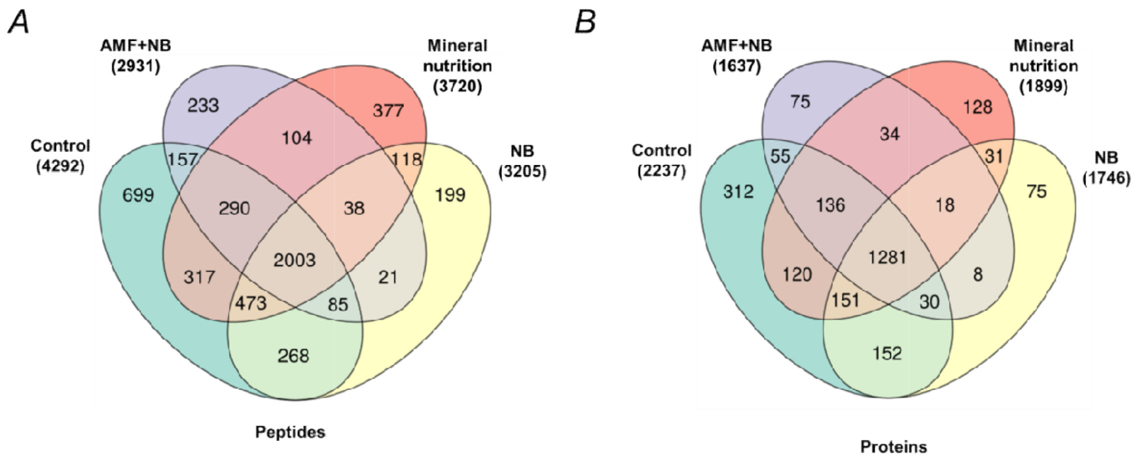

Figure S1-3 The numbers of peptides (A) and proteins (B) identified in the *P. sativum* genotype k-8274 plants grown under different soil supplementation conditions. AMF+NB – plants grown in the presence of arbuscular micorrhiza and nodule bacteria; NB - plants grown in the presence of nodule bacteria; mineral nutrition – plants grown in the presence of mineral salts; control – intact plants.

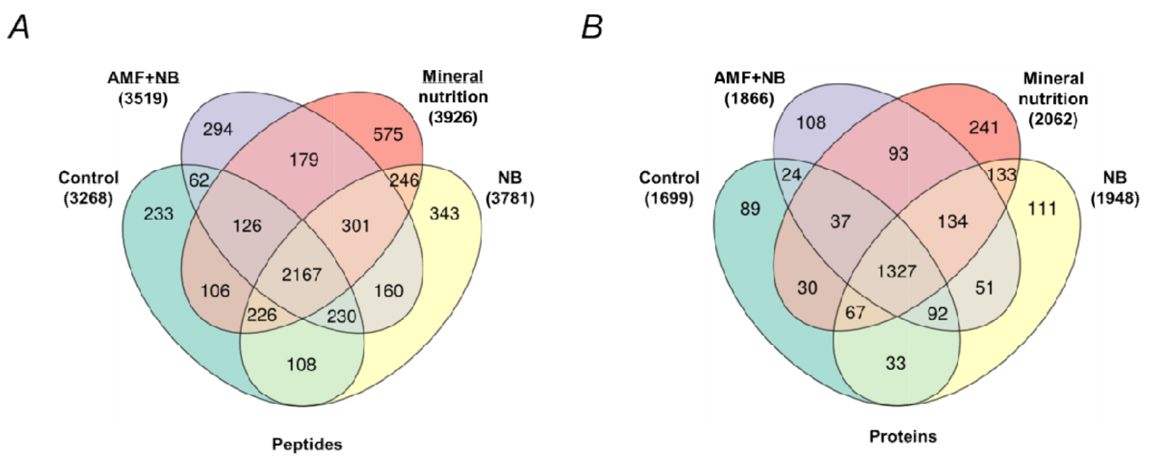

Figure S1-4 The numbers of peptides (A) and proteins (B) identified in the *P. sativum* genotype k-3358 plants grown under different soil supplementation conditions. AMF+NB – plants grown in the presence of arbuscular micorrhiza and nodule bacteria; NB - plants grown in the presence of nodule bacteria; mineral nutrition – plants grown in the presence of mineral salts; control – intact plants.

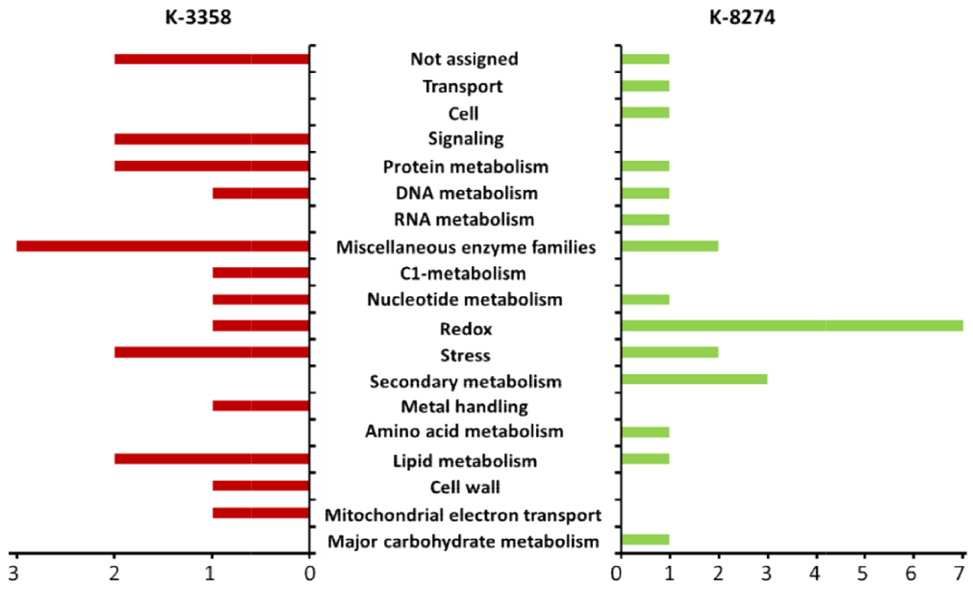

Figure S1-5 Functional annotation of the proteins, which are more abundant in the roots of the pea k-3358 (left, red) and k-8274 (right, green) plants. Numerical values indicate the numbers of proteins constituting individual functional classes (bins).

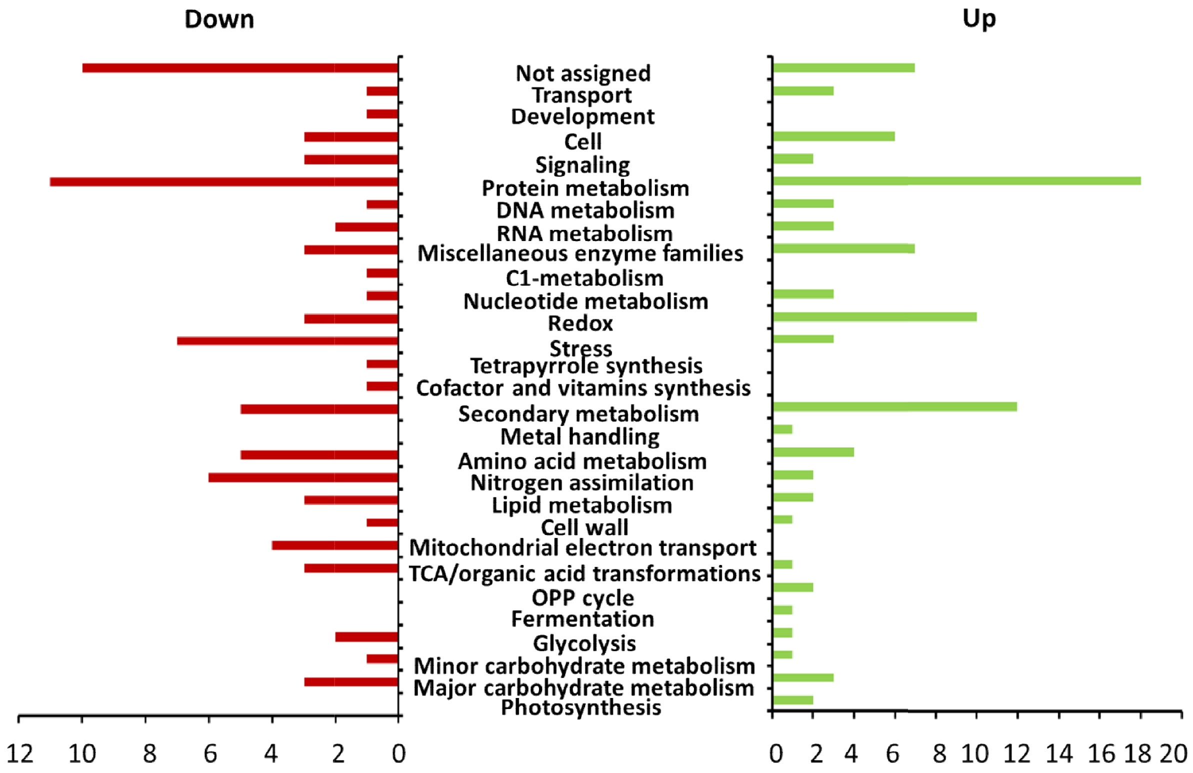

Figure S1-6 Functional annotation of the proteins identified as differentially abundant in the roots of pea (*P.sativum*, genotype k-3358) plants grown without soil complements and in presence of mineral salts supplemented to the soil. Numerical values indicate the numbers of proteins constituting individual protein classes (bins), which were up- (green, right) and down-regulated (red, left) in the presence of mineral soil complementation.

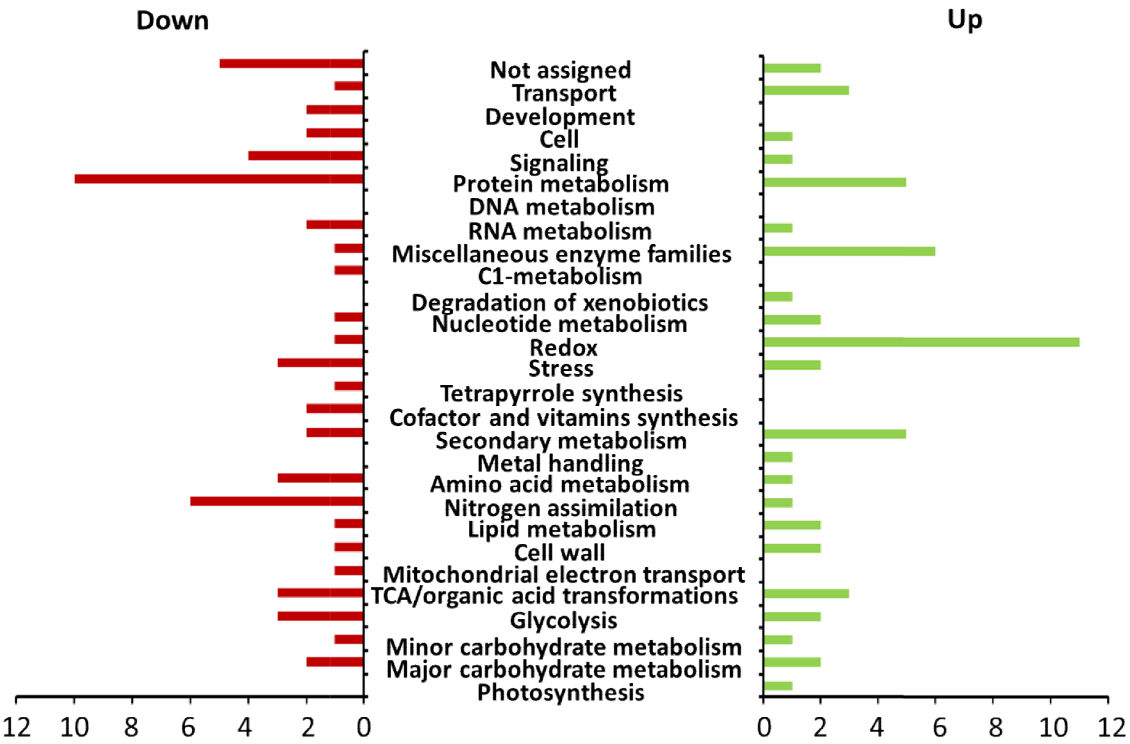

Figure S1-7 Functional annotation of the proteins identified as differentially abundant in the roots of pea (*P.sativum*, genotype k-8274) plants grown without soil complements and in presence of mineral salts supplemented to the soil. Numerical values indicate the numbers of proteins constituting individual protein classes (bins), which were up- (green, right) and down-regulated (red, left) in the presence of mineral soil complementation.

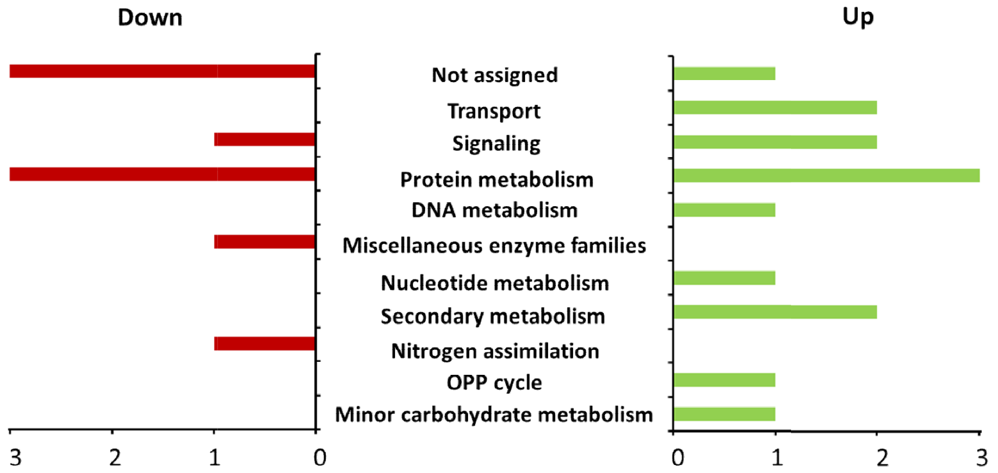

Figure S1-8 Functional annotation of the proteins identified as differentially abundant in the roots of pea (*P.sativum*, genotype k-3358) plants grown without soil complements and in presence of rhizobial bacteria supplemented to the soil (after formation of legume-rhizobial symbiosis, NB). Numerical values indicate the numbers of proteins constituting individual protein classes (bins), which were up- (green, right) and down-regulated (red, left) in the presence of legume-rhizobial symbiosis.

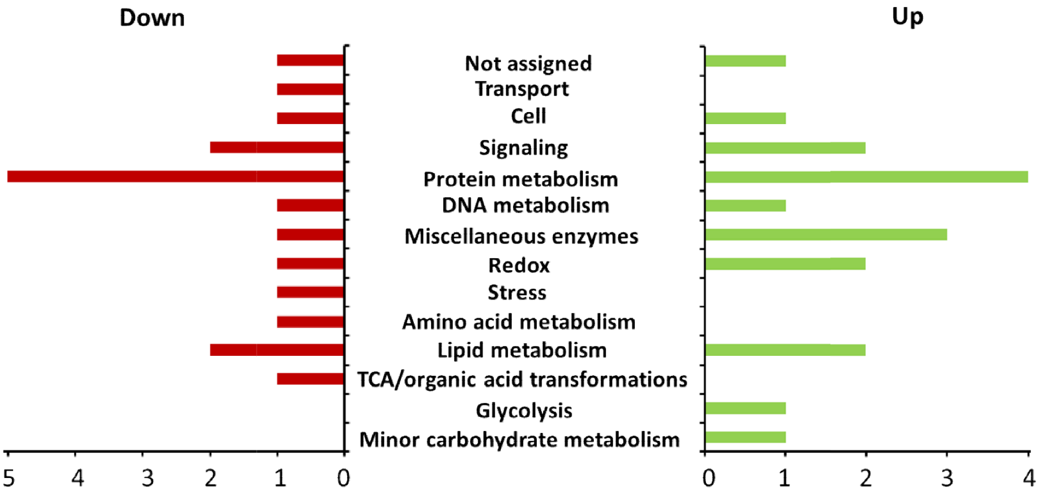

Figure S1-9 Functional annotation of the proteins identified as differentially abundant in the roots of pea (*P.sativum*, genotype k-3358) plants grown without soil complements and in presence of rhizobial bacteria and arbuscular micorrhiza fungi supplemented to the soil (after formation of legume-rhizobial symbiosis and arbuscular micorrhiza, combined inoculation, AMF+NB). Numerical values indicate the numbers of proteins constituting individual protein classes (bins), which were up- (green, right) and down-regulated (red, left) upon the combined inoculation with rhizobia and arbuscular micorrhiza fungi.

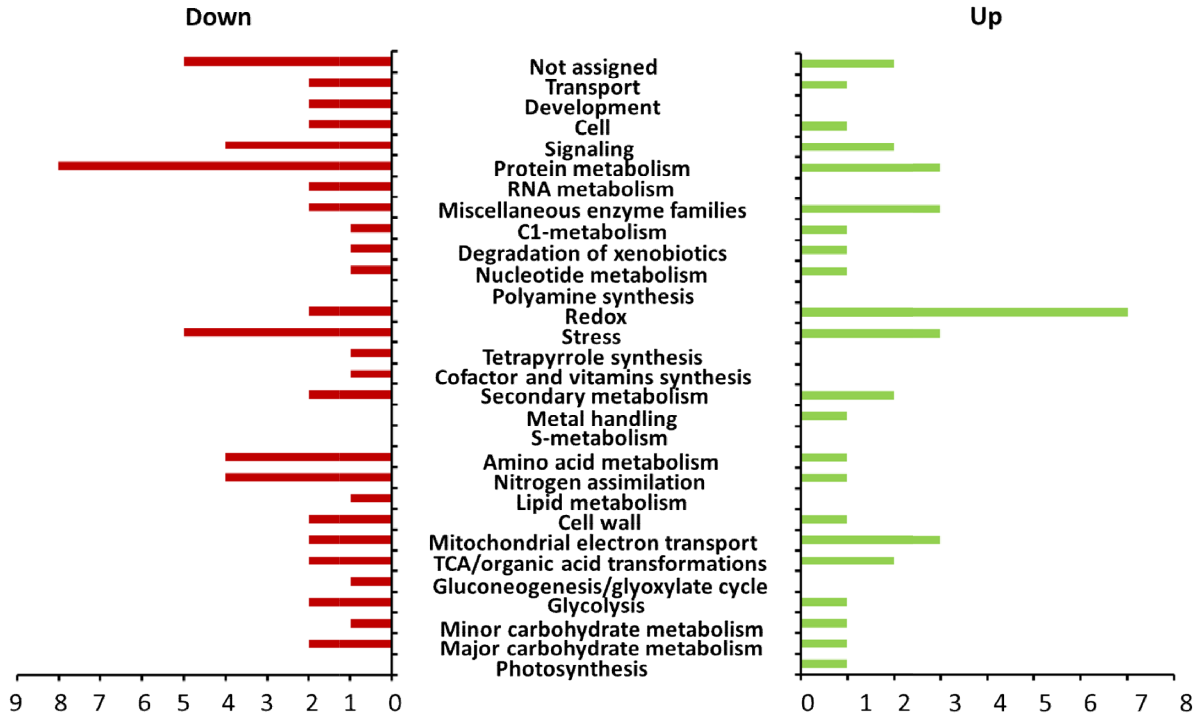

Figure S1-10 Functional annotation of the proteins identified as differentially abundant in the roots of pea (*P.sativum*, genotype k-8274) plants grown without soil complements and in presence of rhizobial bacteria and arbuscular micorrhiza fungi supplemented to the soil (after formation of legume-rhizobial symbiosis and arbuscular micorrhiza, combined inoculation, AMF+NB). Numerical values indicate the numbers of proteins constituting individual protein classes (bins), which were up- (green, right) and down-regulated (red, left) upon the combined inoculation with rhizobia and arbuscular micorrhiza fungi.
